# P-28. A Clinical and Economic Comparison of Non-Egg Influenza Vaccines in Adults 18-64 Years in the U.S

**DOI:** 10.1093/ofid/ofae631.235

**Published:** 2025-01-29

**Authors:** Myron J Levin, Neda Al Rawashdh, Liliane Mofor, Pablo Anaya, Richard M Zur, Emily B Kahn, Daniel Yu, Joaquin F Mould-Quevedo

**Affiliations:** University of Colorado Denver School of Medicine, Aurora, Colorado; IQVIA, Falls Church, Virginia; CSL Seqirus USA, Summit, New Jersey; IQVIA, Falls Church, Virginia; IQVIA, Falls Church, Virginia; IQVIA, Falls Church, Virginia; CSL Seqirus Australia, Melbourne, Victoria, Australia; CSL Seqirus Inc., Summit, New Jersey

## Abstract

**Background:**

Influenza vaccination with non-egg-based vaccines had improved the antibody response to circulating flu viruses over that of traditional egg-based vaccines, which are the most common flu vaccines worldwide. Two non-egg-based flu vaccines— the cell-based flu vaccine (QIVc) and the recombinant flu vaccine (QIVr) —were introduced in the United States during the 2013-14 flu season. The objective of this analysis was to determine the cost-effectiveness of QIVc compared to QIVr administered to people between 18-64 years of age from U.S. commercial and societal perspectives.

**Table 1.**
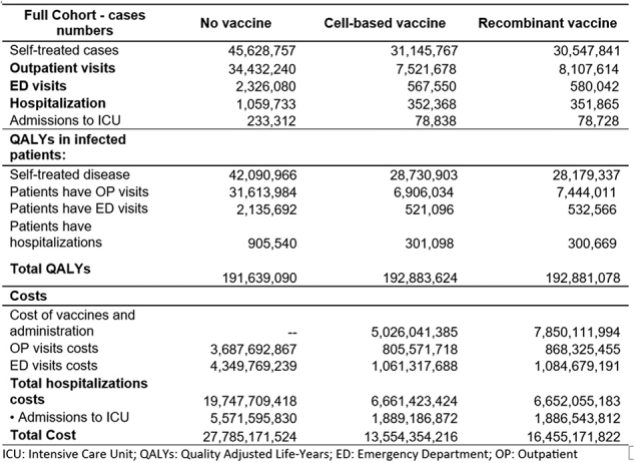
Summary of Health and Economic Outcomes from the Commercial Perspective (Season 2018-19 – Vaccine Effectiveness)

**Methods:**

A compartmental dynamic transmission model, calibrated to match infection data from the U.S., was used to estimate the clinical and economic impact of vaccination over one influenza season. Epidemiological data and vaccination coverage rate were obtained from CDC official databases. Vaccine effectiveness data against outpatient visits and hospitalizations, were obtained from published U.S. observational studies including outpatient and hospital setting outcomes (2018/19 and 2019/20 seasons). Associated direct medical costs, health state utilities and disutilities, and productivity loss costs were obtained from recent economic literature. Deterministic and probabilistic sensitivity analysis were conducted.

**Results:**

From the U.S. commercial perspective of an equal number of vaccinated adults in both groups, QIVc compared to QIVr showed fewer outpatient visits (585,936) and emergency department visits (12,492), and a comparable number of hospitalizations (503) (see Table). QIVc compared to QIVr resulted in a higher overall number of quality-adjusted life years (QALYs) gained (2,546) and a reduction in total associated costs by US$2.9B. Cost savings were driven mainly by the lower 2023/24 acquisition costs, for QIVc vs QIVr (≈-50%). All sensitivity analyses produced similar results, confirming the robustness of the model. Results were consistent from the societal perspective.

**Conclusion:**

The cell-based vaccine was cost saving compared to the recombinant vaccine for subjects 18-64 years in the U.S. and achieved comparable health outcomes results at a significant reduction in cost.

**Disclosures:**

**Myron J. Levin, MD**, CSL Seqirus USA: Advisor/Consultant|Curevo: Advisory Board|GSK: Grant/Research Support|GSK: Advisory Board|Moderna: Advisory Board|Pfizer: Advisory Board **Neda Al Rawashdh, n/a**, CSL Seqirus USA: Advisor/Consultant **Liliane Mofor, n/a**, CSL Seqirus USA: Stocks/Bonds (Private Company) **Pablo Anaya, n/a**, CSL Seqirus USA: Advisor/Consultant **Richard M. Zur, n/a**, CSL Seqirus USA: Advisor/Consultant **Emily B. Kahn, n/a**, CSL Seqirus USA: Advisor/Consultant **Daniel Yu, n/a**, CSL Seqirus Australia: Stocks/Bonds (Private Company) **Joaquin F. Mould-Quevedo, PhD**, CSL Seqirus USA: Stocks/Bonds (Private Company)

